# Evaluating the Effectiveness of an App-Based Nurse-Moderated Program for New Mothers With Depression and Parenting Problems (eMums Plus): Protocol for a Pragmatic Randomized Controlled Trial

**DOI:** 10.2196/11549

**Published:** 2019-01-16

**Authors:** Alyssa CP Sawyer, Amy L Kaim, Christy E Reece, Denise McDonald, Huynh-Nhu Le, Jennifer Clark, John W Lynch, Michael G Sawyer

**Affiliations:** 1 School of Public Health University of Adelaide Adelaide Australia; 2 School of Medicine University of Adelaide Adelaide Australia; 3 Research and Evaluation Unit Women’s and Children’s Health Network North Adelaide Australia; 4 Child and Family Health Service Women’s and Children’s Health Network Adelaide Australia; 5 Department of Psychology George Washington University Washington, DC United States; 6 Population Health Sciences University of Bristol Bristol United Kingdom

**Keywords:** app, infant, mobile phone, mother-child relations, mother-child nursing, postnatal depression, protocol, randomized controlled trial

## Abstract

**Background:**

Postnatal depression adversely affects many mothers and infants with good evidence that caregiving difficulties associated with depressive symptoms play a key role in later adverse childhood outcomes. In many countries, there is only limited support available for women who experience symptoms of depression during the postnatal period, particularly those experiencing subthreshold symptom levels. Furthermore, mental health services and community family health services in many countries tend to focus primarily on providing help for depressive symptoms or maternal caregiving, respectively, despite these problems commonly being comorbid. Group-based nurse-led interventions delivered over the Web through mobile phone “apps” have the potential to be a cost-effective method of providing a large number of mothers with easy access to integrated support for both maternal depressive symptoms and caregiving difficulties.

**Objective:**

This paper describes the protocol for a pragmatic randomized controlled trial of a 4-month group-based nurse-led intervention delivered over the Web when infants were 2-6 months. The primary aims of the trial are to determine whether the intervention (1) reduces levels of maternal depressive symptoms and (2) improves the quality of maternal caregiving when infants are 8-12 months of age.

**Methods:**

The trial aimed to recruit and randomize 160 mothers of infants aged 2-8 weeks to either the intervention (eMums plus) or standard care. Assessments were completed when infants were aged 1-2 (preintervention), 8, and 12 months. The primary outcomes were the level of maternal depressive symptoms and the quality of maternal caregiving assessed when infants were aged 12 months. The intervention provided specific support for problems with mood and problems with caregiving. The intervention was delivered by community health nurses as a part of routine service delivery to mothers via a mobile phone app.

**Results:**

Participant recruitment was carried out from March to July 2017. Follow-up data collection was completed in mid-2018. Data analysis has commenced.

**Conclusions:**

In the past, many mothers participated in nurse-led face-to-face groups postnatally. However, mothers’ groups held in clinics can be difficult for busy mothers to attend. The eMums intervention was delivered over the Web by nurses, allowing easy access by mothers early in an infant’s life. The intervention was evaluated while delivered as part of the routine service practice by community child health nurses. The advantage of evaluating the effectiveness of the intervention in the routine service practice is that if it is found to be effective, it can be more easily adopted by the service provider than if it had been assessed in an efficacy trial.

**International Registered Report Identifier (IRRID):**

RR1-10.2196/11549

## Introduction

### Background

Postnatal depression is associated with a range of adverse child outcomes [1-3]. Furthermore, there is good evidence that caregiving difficulties associated with depressive symptoms play a key role mediating the association between maternal depression and adverse child outcomes [4-6]. A range of important caregiving practices are adversely affected by postnatal depression, including breastfeeding, sleep routines, health check attendances, and vaccinations [4]. Problems in all of these areas can adversely affect children’s longer-term growth and development. The United Kingdom’s National Institute of Clinical Excellence guidelines for ante- and postnatal care highlight that even subthreshold depressive symptoms can adversely affect mothers’ general functioning and infant development [7]. This has led many countries, including Australia, to initiate universal screening programs to identify mothers with depressive symptoms [8]. However, a major ongoing challenge is the very limited availability of support services for mothers who screen positive and difficulty engaging busy new mothers with clinic-based treatment programs [8].

In 2014, a new National Institute of Clinical Excellence guideline specifically advocated for randomized controlled trials (RCTs) focused on mothers experiencing subthreshold depressive symptoms to test the effectiveness of interventions designed to improve mother-baby relationships in this large group of mothers [7]. In the Australian national postnatal depression screening program, 7.5% of women scored >12 on the Edinburgh Postnatal Depression Scale (EPDS), suggesting the presence of postnatal depression, and 8.0% scored in the range of 10-12 on EPDS [9]. The latter is important as subthreshold levels of postnatal depression symptoms in the early postnatal period are a risk factor for the development of postnatal depression and also have the potential to interfere with optimal mother-infant development [6,10,11]. The aim of this trial is to evaluate a new app-based intervention designed to help mothers experiencing postnatal depressive symptoms and parenting problems. The intervention employed mobile phone technology to provide easy access for a large number of new mothers to both nurse and peer support during the immediate postnatal period.

Two broad approaches have been used to help mothers with symptoms of depression and parenting problems. Commonly, these approaches are delivered in separate services and provide support for either depression or parenting problems. First, community child and family health services provide help that focuses on (1) improving maternal parenting skills and self-efficacy; (2) reducing mother-infant problems; and (3) supporting maternal health and well-being. A significant strength of community services is that they have direct contact with a very high proportion of all mothers during the immediate postnatal period. As such, they are well placed to screen mothers for the presence of problems and help a large number of mothers with difficulties in these areas. However, the help provided by these services is limited and largely focuses on caregiving problems rather than maternal depressive symptoms. Additionally, nurse training and confidence needed to manage maternal postnatal depression is often limited. The second approach, which is widely used in mental health services, typically employs psychosocial programs based on the cognitive behavioral therapy or interpersonal psychotherapy [7]. However, an important limitation of these programs is that, in contrast to child and family services, they largely focus on maternal depressive symptoms rather than caregiving problems [6]. Furthermore, mental health services often lack the resources to provide help to those with subthreshold levels of depressive symptoms, reserving treatment for those with serious mental illness. As a result, mothers with subthreshold depressive symptoms can have little or no access to professional services. Postnatal depressive symptoms also have unique triggers, such as the challenges of the transition to a parenting role, time demands required to care for new infants, and the potential for social isolation during this period. Given this, it is not surprising that there is little evidence that current mental health programs have a positive effect on mother-infant problems or longer-term child outcomes [12].

The intervention tested in this trial was based on the evidence that maternal self-efficacy and social support are two key mechanisms influencing the onset, maintenance, and impact of maternal postnatal depressive symptoms [13,14]. Perceptions of self-efficacy influence the extent to which individuals feel that they can cope with demanding life situations, and this, in turn, can shape affective responses to stressful role changes such as becoming a new mother [15]. For example, mothers who lack confidence in their ability to settle their distressed infant are more likely to give up quickly, leading to a sense of failure and depressed mood [14]. They are then more likely to experience persistent infant problems, such as feeding and sleeping difficulties, placing them at greater risk for elevated depressive symptoms. Additionally, social support appears to play a protective function in the postnatal period by reducing the stress associated with the transition to motherhood [14]. However, greater family mobility, changes to female participation in the labor market, and increasing time pressures on young families have made access to traditional sources of family and professional support more difficult. These increase the risk that new mothers will become socially isolated and lack daily support [16]. As such, app-based interventions that facilitate easy access to social and professional support may be particularly important for new mothers experiencing depressive symptoms and struggling with parenting demands [17].

In order to support a large number of women who experience subthreshold levels of depression, new approaches are needed that can help a larger number of mothers than it is possible to support using traditional face-to-face programs. Web-based interventions are one way through which this can be achieved. Internet access among women of child-bearing age in Australia is now ubiquitous, with new mothers making extensive use of the internet to obtain child-raising information and social support. This has encouraged the development of numerous websites and “phone apps” by commercial, professional, and government organizations. However, health-related information on the internet can often be misleading and, occasionally, “utterly wrong” [16]. As well, there is a marked absence of evaluations assessing the extent to which Web-based information and support is utilized by mothers and improves maternal and child outcomes.

The intervention tested in this trial was based on our previous evaluation of “eMums,” an innovative group-based nurse-led intervention delivered over the Web that provided support for common parenting difficulties for the general population of mothers [18,19]. The intervention tested in this trial “eMums plus” builds on this work, with the addition of integrated support for depression as well as parenting difficulties. The eMums plus intervention was based on 4 core principles. First, it was designed to provide both peer and professional support as there are a number of Web-based interventions that provide information alone without peer or professional support but when tested have very low usage rates [20,21]. Second, it was designed as an app-based intervention to enable easy access very early after birth without requiring travel or attendance at clinics at specific appointment times. Third, it was designed to have the capacity to support a larger number of mothers than is possible with face-to-face care. Finally, it was designed and tested in a pragmatic RCT in collaboration with a statewide Child and Family Health Service (CaFHS). This was done to ensure that the intervention was readily translatable into standard clinical practice in contrast to efficacy trials where interventions are generally developed and tested by researchers in academic settings and then have to be adapted to the demands of routine clinical service. To the best of our knowledge, no previous study has evaluated the effectiveness of a group-based nurse-led intervention delivered over the Web through routine services and designed to reduce maternal depressive symptoms and parenting problems.

### Objectives and Hypotheses

The protocol for the study utilized an RCT to determine whether a 4-month group-based nurse-led intervention delivered over the Web through a mobile phone app when infants were 2-6 months reduced levels of maternal depressive symptoms and improved the quality of maternal caregiving when infants were aged 8-12 months.

We hypothesized that when their infants are aged 12 months, questionnaire scores and direct observation assessments would indicate that mothers who received the app-based intervention would be functioning better than comparison mothers with (1) lower scores on the EPDS [22] assessing the level of maternal depressive symptoms; (2) higher scores on the Parenting Sense of Competence Scale (PSCS) score assessing mothers’ perception of their maternal caregiving competence [23,24] and lower scores on the Parenting Stress Index (PSI) [25] Competence subscale indicating the improved sense of competence in caregiving; and (3) higher scores on the Nursing Child Assessment Satellite Training (NCAST) Parent-Child Interaction Teaching Total Scale scores [26], assessing the quality of mother–child interactions, and lower scores of the PSI Attachment subscale indicating the improved parent-child relationship quality.

## Methods

### Study Design

The study was a pragmatic RCT of a group-based nurse-led intervention delivered over the Web versus “standard care.” The trial was embedded into the routine service practice in the statewide CaFHS. This enabled the trial to examine whether the intervention was effective when delivered as a part of the routine service delivery [27,28].

### Setting

Participants were recruited from 14 CaFHS sites located in major urban areas and a large regional center in South Australia. CaFHS is the key community health service in the State, providing a range of services for mothers and infants, including infant health checks and home-based maternal support.

### Participants and Recruitment

**Figure 1:**
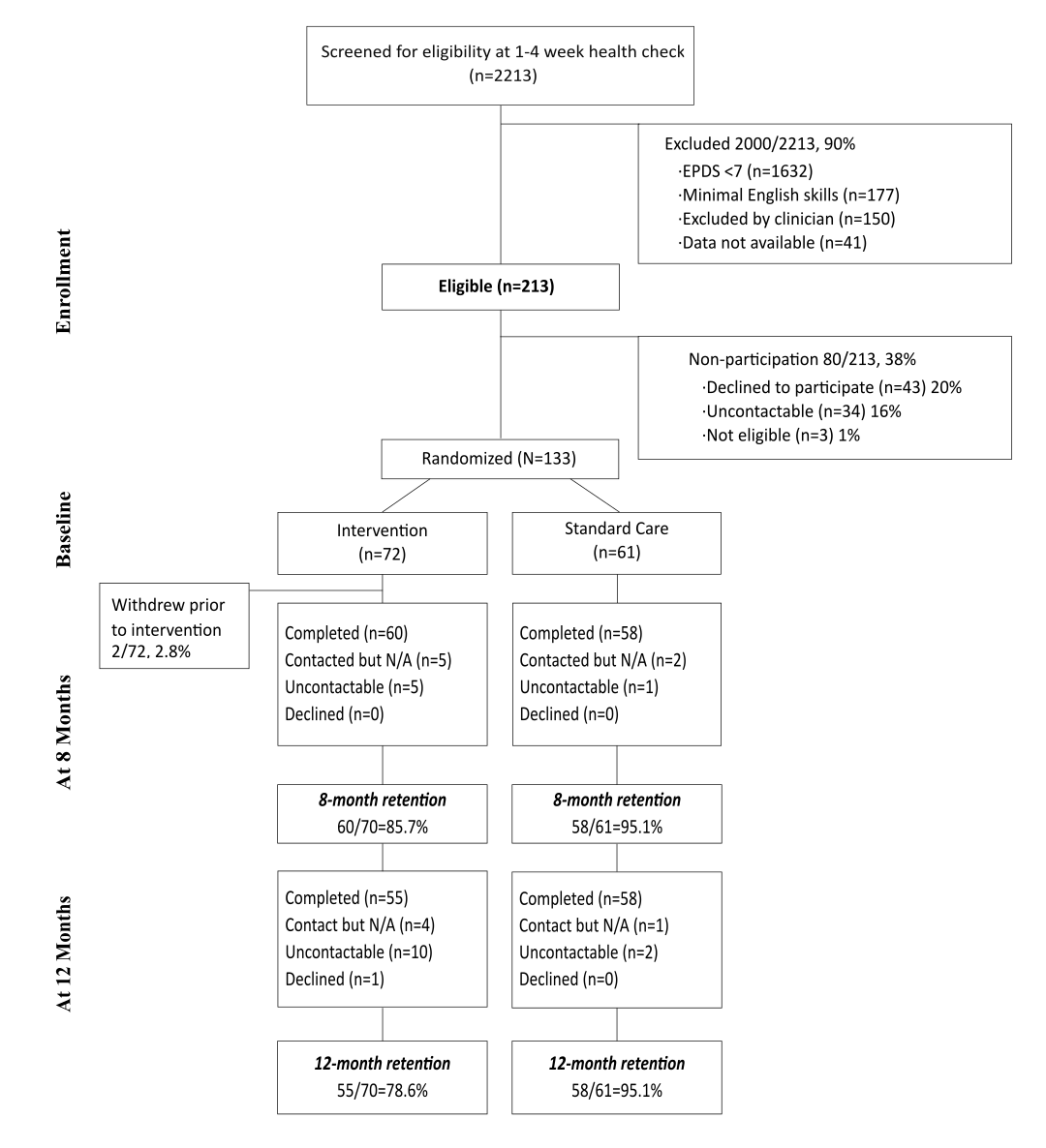
Flow chart of participants. EPDS: Edinburgh Postnatal Depression Scale; N/A: not available.

We aimed to enroll 160 mothers of infants aged 2-8 weeks at the time that they completed their 1-4-week postnatal health check with CaFHS (Figure 1). At the time CaFHS administration staff routinely made contact with mothers to organize their postnatal health check, potential participants were asked whether they were willing to consider participating in the research project should they prove to be eligible for the study. Mothers who indicated that they were willing to consider participation were asked to consent to allow their phone contact details to be passed on to the research team, if they were eligible for the study. During the postnatal health check, these mothers completed a questionnaire comprising a 4-item parenting problems scale and the EPDS [22]. Mothers who scored >7 on the EPDS and reported, at least, 1 problem on the 4-item parenting problems questionnaire were eligible to participate. Following their postnatal health check, eligible mothers were contacted by telephone by the research team. The research team explained the study to the mother, sought verbal consent for a home visit by a member of the study’s field-worker team, and randomized mothers to either the intervention arm or the comparison (“standard care”) arm of the study. Field workers contacted mothers who had given their consent and arranged a time to visit them to complete the “formal” consent process and, where written consent was given, complete the baseline research assessment.

The exclusion criteria included mothers (1) with an EPDS score >13 and who were judged by their screening nurse to have a level of depressive symptoms that precluded their participation in the study; (2) those judged by their screening nurse to be experiencing domestic violence, illicit drug use, or other major distress that precluded their participation in the study; or (3) those who lacked sufficient English skills to complete the self-report questionnaires. Mothers with an EPDS score >13 whose nurses judged they would be able to participate in the trial were included provided they also had access to support from a family doctor or other health professional. All mothers identified as experiencing high levels of depressive symptoms (ie, >13 on the EPDS) were referred to other services, most frequently general practitioners. Such referrals occurred in both the intervention and comparison groups in this study.

Following randomization, mothers in the intervention arm were assigned to a group over the Web supported by a CaFHS nurse comprising approximately 20 mothers of similar-aged infants; they were also able to access standard care services as required. The intervention was delivered when infants were aged 2-6 months because this is (1) a key developmental period for infants; (2) a time when mothers are most vulnerable to depressive symptoms; and (3) as previously shown, it is the time when mothers most actively seek information from nurses and want to share and exchange ideas with each other [18].

### Randomization

The trial used group randomization with blocks of 20 eligible mothers consecutively identified and then randomized to either the intervention or comparison arms of the study. A group randomization sequence was used to determine the arm to which each group was assigned. This approach ensured that mothers’ groups in the intervention arm contained no more than 20 mothers per group. The randomization schedule was generated by a statistician who was independent of the study team. The research team was blind to group allocation at the time of recruitment and assignment of mothers to the study groups. However, due to the nature of the intervention, after the intervention commenced, it was not possible to keep research staff or field workers blind to the groups to which mothers had been allocated. The exception to this was research staff members coding the NCAST Parent-Child Interaction scale, who were blind to the group allocation while completing coding.

### Intervention Delivery

The intervention comprised a 4-month group-based nurse-led program delivered over the Web by community health nurses to mothers via a mobile phone app when their infants were 2-6 months of age. A key premise of the intervention was that to be effective, help for new mothers experiencing problems with their mood and caregiving role must be closely integrated. To achieve this, the intervention was designed to (1) reduce maternal depressive symptoms; (2) support mothers to gain competence and self-efficacy in caring for their infants and solving caregiving difficulties; and (3) support mothers to achieve healthy lifestyles for themselves and their infants.

Nurses who delivered the intervention received training in the use and management of the app from the lead nurse who was extensively involved in the delivery of our original eMums program and from the research team. Nurses also received an additional 3 days of training in the delivery of the mental health components of the eMums plus intervention and general training in responding to those experiencing mental health problems (outlined below).

**Figure 2:**
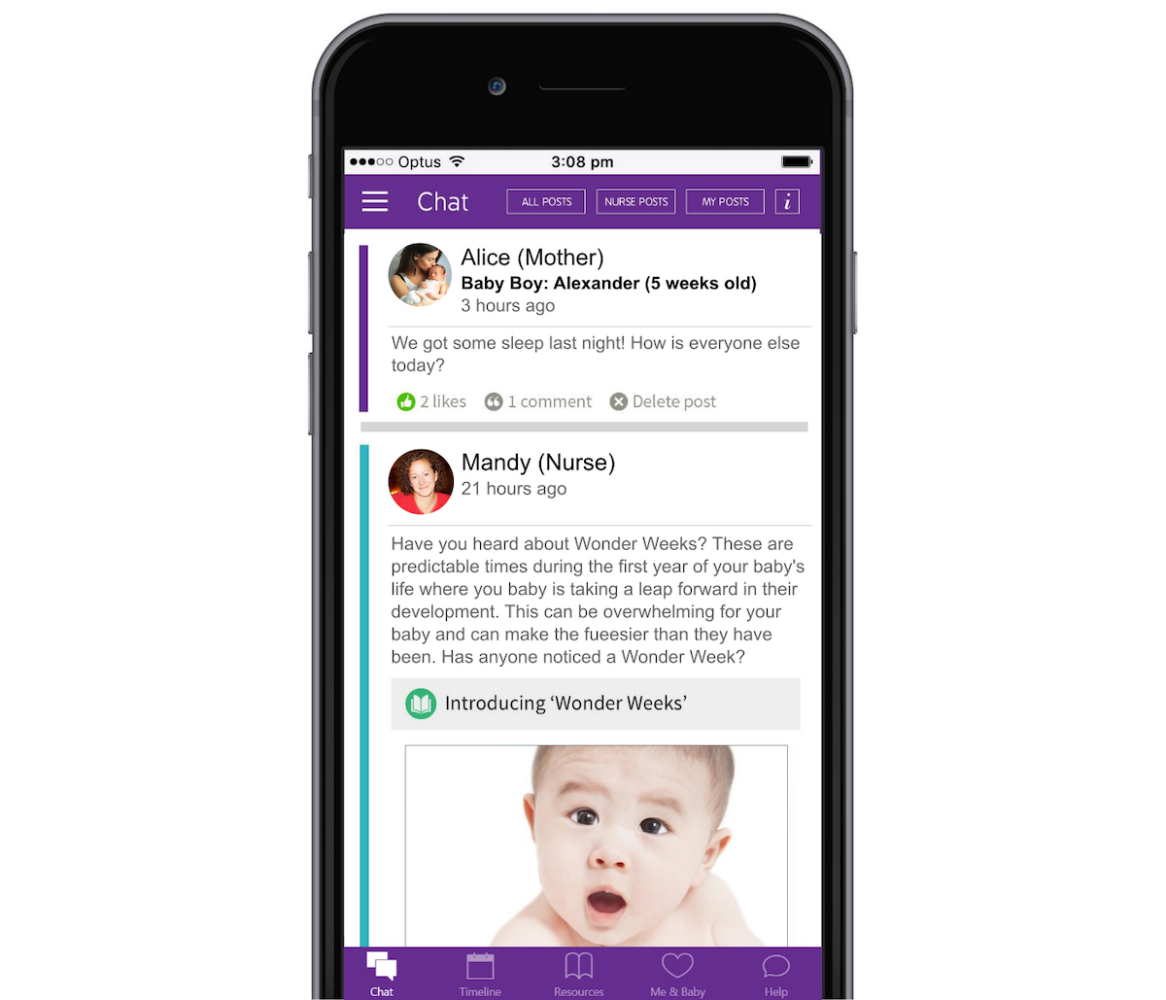
The eMums plus mobile phone app. (Source: image taken from eMums plus intervention).

The “mother’s view” of the app comprises 4 components, each of which is explained in an “Orientation” given at the beginning of the program (see Figure 2).

The components are highlighted in a “site-map” available with the app. The 4 components for mothers are as follows:

1. *Chat:* It contains a chat room where mothers post questions and nurses can reply with posts and comments visible to all group members in a similar format to Facebook. Mothers can also reply and answer each other’s questions. The parenting and emotional health curriculum is posted on the chat room 2 times a week for mothers. Furthermore, the nurse posts additional content depending on the needs of her group.

1. *Timeline:* It provides a list of child development milestones and health reminders that provides guidance to mothers appropriate to their baby’s age during the intervention. Mothers can record these items as “completed” on the eMums plus app. Nurses can also view the timeline to assess whether children have completed health checks and are meeting developmental milestones. Mothers can also access a maternal and infant “mood-rater” that allows mothers to monitor their own mood and nurses to track mothers’ and infants’ moods over time. It also contains an events calendar displaying topics that nurses discuss and other material relevant to the functioning of the group.
2. *Resources:* It contains short articles and activities on parenting and emotional health that make up the eMums plus curriculum, as well as additional information about other topics that may be useful for mothers. This is available for mothers to search as required if they are looking for accurate CaFHS-endorsed information on a particular topic. Mothers are able to post topics from the resources section into the chat page if they want to share information with the group.
3. *Contacts and Assistance:* It contains useful contact numbers and a portal through which mothers can privately message their group’s nurse. Nurses are able to respond to mothers privately and send messages and notifications about upcoming discussions to all the mothers in their group.

The “nurses’ view” of the app comprises 3 main elements as follows:

1. *Group Dashboard:* It displays information about individual groups such as group activities, notes maintained by nurses, and responses to mood ratings completed by mothers.
2. *Parent Dashboard:* It displays information about individual mothers, including parent case notes, individual website log-in activities (eg, when mothers view material, such as a depression module or the content of the chat room, mothers’ latest ratings of their mood, and their babies’ mood), and notifications that mothers have added information about children’s milestones.
3. *Nurse Home Group:* It enables nurses to access their group’s chat room and contains additional resources that nurses utilize (eg, information inserted into the group chat room such as messages, reminders, and curriculum topics). In addition to accessing the program on their computer, nurses also have a nurse-app installed on their mobile phones. This app contains all the features of the mothers’ app, with additional capabilities that allow them to send messages and notifications about upcoming discussions to all the mothers in their group. At the beginning of the program, nurses welcome mothers to their group and outline the goals of the program. They explain how to make the best use of the app and its various features (eg, the use of notifications to mothers about when the nurse will be online and topics scheduled for discussion).

### Intervention Content

The intervention has 2 main curriculum components and a training component for nurses delivering the intervention. The curriculum consists of information provided to support mothers with parenting problems and a modulized program designed to reduce symptoms of depression.

#### Maternal Parenting Problems

For the eMums plus program, we have adapted existing CaFHS parenting resources to create an app-based intervention designed to help mothers with parenting during their infants’ first months of life. The curriculum focuses on improving maternal caregiving through anticipatory guidance about infant development, problem solving, common parenting difficulties, promoting maternal sensitivity and responsiveness, and providing social support. The parenting content includes steps that mothers can take (1) to resolve common practical problems experienced by mothers of young children (eg, feeding, sleeping, and “settling”) and (2) to look after their own health and well-being. It also shows mothers’ activities that they can use to promote the health and development of their infants (eg, improving parent-infant attachment and stimulating infant language development). The curriculum is comprised of weekly modules containing information and links to further information in the “Resources” section of the website. This section can be accessed independently by mothers. While the curriculum has a schedule for delivery, nurses use their experience of child development and maternal psychosocial role adjustment, as well as the content of mothers’ discussions and questions on the Web, to tailor the curriculum to each group’s particular needs. This “just-in-time” approach is used because clinical experience suggests that new mothers primarily want information relevant to their specific situation and the age of their infant, rather than more broadly based anticipatory advice about what might occur in the future.

#### Maternal Depressive Symptoms

The curriculum addressing maternal depressive symptoms is adapted from the *Mothers and Babies Course*, a manualized group treatment program for postnatal depression [29,30]. The *Mothers and Babies Course* is an evidence-based program that has been demonstrated to reduce maternal depressive symptoms in efficacy trials [29,30]. Our adaptation of the *Mothers and Babies Course* makes it appropriate for delivery in combination with the parenting intervention content. The program is based on the cognitive behavioral theory and attachment theory and targets the unique needs and stressors mothers face during the postnatal period. It has achieved promising results in initial trials delivered alone and in combination with a home-visiting program provided to low-income mothers. In the app, module content is presented in the form of text and video or audio messages. Each module provides mothers with a task to complete (eg, behavioral activation), with the aim of prompting group discussion and problem solving around the task, and engaging mothers in managing mood and the stressors of parenthood. The timing of the presentation of depression modules is based on children’s ages, and they are largely “self-help” rather than requiring nurse assistance for their completion. The primary role of nurses is to encourage the use of the modules. Nurses receive training in the use of their content from a clinical psychologist trained in the delivery of the *Mothers and Babies Course*.

#### Mental Health First Aid

All nurses delivering the intervention completed the *Mental Health First Aid* [31,32] training program with the aim of improving their ability to identify depressive symptoms and provide support for mothers experiencing symptoms of postnatal depression. This training program was designed to help lay individuals and professionals develop skills in working with individuals developing mental health problems or in a mental health crisis. A meta-analysis of trials evaluating *Mental Health First Aid* has shown that this training increases participants’ knowledge regarding mental health, decreases their negative attitudes, and increases supportive behaviors for individuals with mental health problems [33].

The use of these curriculum and training components ensured that the intervention appropriately addressed both maternal depressive symptoms and problems with parenting.

### Standard Care

Only mothers in the intervention arm had access to the internet intervention. However, mothers in both intervention and comparison groups had access to standard care arrangements.

For the vast majority of mothers, standard care comprised a single home visit by a CaFHS nurse who checks the health of mothers and infants, provides advice about issues relevant to infant care, and offers information about other relevant community services available for mothers and infants.

### Outcome Measures

All measures were completed when infants were aged 1-2 (preintervention), 8, and 12 months. Measures were completed during home visits conducted by trained field workers to ensure high-quality data. The primary outcomes for the trial were the level of maternal depressive symptoms and observed the quality of maternal caregiving assessed when infants were aged 8 and 12 months.

#### Maternal Depressive Symptoms

The EPDS is a 10-item self-report questionnaire that assesses the level of depressive symptoms experienced by mothers during the postnatal period [22]. Questions assess symptomatology during the previous 7 days and utilize a 4-point response scale and include items such as *“I have blamed myself unnecessarily when things went wrong* ” and “*Things have been getting on top of me*.” Scores on all items are summed and recommended cutoff points are available to identify mothers who would benefit from additional support [34]. Scores range from 0 to 30, with higher scores indicating higher levels of depression symptoms.

#### Maternal Caregiving

##### The Parenting Sense of Competence Scale

PSCS is a 16-item self-report questionnaire designed to measure the parental efficacy and satisfaction in the parenting role. Items are rated on a 6-point response scale and include “*Being a parent is manageable, and any problems are easily solved* ” and “*Being a parent makes me tense and anxious*.” Scores range from 16 to 96, with higher scores indicating higher levels of parenting competence. The scale has been successfully used with Australian mothers and has satisfactory psychometric properties [23,24].

##### Nursing Child Assessment Satellite Training Scales

NCAST scales are designed to assess the quality of mother–child interactions, including the sensitivity to cues, response to distress, fostering social–emotional functioning, and fostering cognitive growth [26]. For the purpose of this study, we used the Teaching Scale suitable for use with 0-36-month olds. The scale utilizes 3-5-minute video-recordings of mothers teaching their child a skill appropriate to the age of their child, selected from a list in the NCAST training manual. Field workers recorded mothers completing the teaching interaction during home visits. Subsequently, research assistants who have completed the NCAST training program coded the video-recordings to generate a total score and subscale scores [26]. Items were coded as “Yes” or “No” and include “*Child attempts to engage caregiver in eye-to-eye contact* ” and “*Caregiver praises child’s successes or partial successes*.” Scores range from 0 to 73, with higher scores indicating higher levels of positive mother–child interaction quality. The NCAST scale has been found to have satisfactory psychometric properties [26].

##### Parenting Stress Index

PSI is a widely used self-report questionnaire designed to assess parent and child characteristics relevant to “parent-child systems” with acceptable psychometric properties [25]. Items consist of statements such as “*I often have doubts about my ability to handle being a parent* ” and “*I expected to have closer and warmer feelings for my child than I do and this bothers me* ” and are rated on a 5-point response scale. Relevant subscales assess maternal perceptions of parenting competence, the quality of parent-child relationships, and the impact of parenting responsibilities on autonomy and self-identity. Higher scores indicate worse functioning with scores for parenting competence (11 items, excluding 2 items assessing parental education) and the quality of parent-child relationships (7 items) with range 11-55 and 7-35, respectively.

#### Other Measures

##### Service Utilization

Service utilization was included as a secondary outcome for this study, as it is possible that mothers who received eMums plus required less support from other services. This information is routinely recorded by CaFHS, including the number of clinic visits and the number of health checks. Maternal self-report questionnaires identified other services (eg, general practitioners) used by mothers and infants.

##### Intervention Quality

Mothers’ perceptions about the quality of the support provided by the intervention were assessed using a 40-item questionnaire after 8 months, which we have developed for this purpose. Items asked about the intervention effectiveness and usability of the mobile phone app. After 12 months, mothers were asked to answer 10 questions about what had been the most useful elements of the intervention.

##### App Usage

The extent to which mothers used the app was recorded. Data were automatically collected on a number of indices, including the number of log-ins, comments, and replies that mothers post, as well as the amount of time spent in different sections of the app.

### Analysis Plan

Analyses will be by intention-to-treat and focus on intervention effects on maternal depression (EPDS) [22], mother–child interactions (NCAST) [26], and maternal caregiving competence (PSI and PSCS) [23,25] at 8 and 12 months. General linear modeling techniques will be used when the scores are continuous outcomes, including log-binomial regression for dichotomous outcomes (eg, the percentage of mothers scoring above recommended EPDS cutoff scores). Data collected at baseline will be used to control for any imbalances between the trial arms. Multiple imputations will be used to address missing data where this approach is appropriate.

### Sample Size

The sample size target for this study was 160 (80 in each trial arm). This sample size would provide 0.80 power to detect an effect size of Cohen *d*=0.4 at an alpha of .05.

### Ethics

Ethics approval was received from the Women’s and Children’s Health Network Human Research Ethics Committee (approval numbers SSA/16/WCHN/016, HREC/16/WCHN/014).

## Results

Participant recruitment was carried out from March to July 2017. Follow-up data collection was completed in June 2018. Data analysis has commenced.

## Discussion

The broad aim of this study was to assess whether a 4-month group-based, nurse-led intervention delivered through a mobile phone app when infants were aged 2-6 months reduced levels of maternal depressive symptoms and improved the quality of maternal caregiving when infants were aged 8-12 months. The intervention was assessed in a pragmatic RCT with the intervention delivered as part of the routine service practice by community child health nurses. The advantage of this methodology is that when an intervention is found to improve child and maternal outcomes services, it can be more readily taken up and continue providing this service using staff already experienced in delivering the intervention. It has been widely recognized in the medical and public health literature that results from such trials are more likely to be translated into practice than results from trials conducted in academic or research settings [27,28].

A large number of mothers experience subthreshold levels of postnatal depression, and it has been recognized that even subthreshold levels of depression can adversely affect maternal functioning and infant development. If the results from the trial demonstrate a positive effect, it will have established the basis for a new app-based approach that can help a large number of mothers experiencing depressive symptoms and caregiving difficulties early in their infant’s life, including mothers in rural communities who frequently have limited access to clinic-based services.

## Abbreviations

CaFHS: Child and Family Health Service
EPDS: Edinburgh Postnatal Depression Scale
NCAST: Nursing Child Assessment Satellite Training
PSCS: Parenting Sense of Competence Scale
PSI: Parenting Stress Index
RCT: randomized controlled trial
